# Unsuccessful Endovascular Treatment in a Patient With Stroke Onset of Takayasu Arteritis and Positive Clinical Outcome

**DOI:** 10.7759/cureus.11980

**Published:** 2020-12-08

**Authors:** Marilena Mangiardi, Maria Cristina Bravi, Francesca Romana Pezzella, Lorenzo Ricci, Sabrina Anticoli

**Affiliations:** 1 Neurology, Azienda Ospedaliera San Camillo Forlanini, Rome, ITA; 2 Stroke Unit, Azienda Ospedaliera San Camillo Forlanini, Rome, ITA; 3 Neurology, Universita' Campus Biomedico, Rome, ITA; 4 Stroke Unit, San Camillo Hospital, Rome, ITA

**Keywords:** stroke, takayasu arteritis, endovascular treatment

## Abstract

Takayasu's arteritis (TA) is a chronic progressive vasculitis affecting large and medium-sized vessels, mainly in young subjects. It is most common in women with a higher prevalence in the Asian population. Stroke is a rare complication of TA, and these patients usually have a poor therapeutic response to revascularization treatments (thrombolysis and/or thrombectomy). We report a case of a male patient aged between 40 and 50 years admitted to our Emergency Department's Stroke Unit for sudden left hemiplegia, hypoesthesia, and dysarthria caused by right internal carotid artery (ICA), middle cerebral artery (MCA), and anterior cerebral artery (ACA) occlusion. He was treated with intravenous thrombolysis (r-tPA), endovascular carotid stenting, and thromboaspiration. We also revealed subclavian stenosis, vascular bruit, erythrocyte sedimentation rate (ESR), and C-reactive protein (CRP) elevation; therefore, a diagnosis of TA was made. Double antiplatelet therapy (DAPT) was started. Despite the early post-procedural carotid stent occlusion, the patient was discharged with a full recovery (neurological index of stroke scale [NIHSS] = 0). Thefive5-year clinical follow-up showed no clinical neurological relapses, and no arterial restenosis was found by further carotid artery echo-Doppler. Takayasu arteritis is a rare cause of ischemic stroke in young adults; however, stroke may be the first manifestation of the disease. Guidelines concerning the role of revascularization treatment in this type of patients are unclear. In this regard, the clinical experience and the multidisciplinary approach applied in our case had a pivotal role. Such an approach would eventually advocate for standardized treatment in patients with stroke and TA.

## Introduction

We present a rare complication of Takayasu’s arteritis (TA) manifesting as an acute ischemic stroke refractory to revascularization treatments. We show the extreme importance of a multidisciplinary approach in the clinical decision-making process for this rare and severe systemic immune-mediated vasculitis presenting with acute stroke. In our case, the successful combination of medical therapy and endovascular approach led to a complete neurological recovery. We advocate for a standardized multidisciplinary approach in all patients with TA manifesting with acute ischemic stroke.

## Case presentation

**Figure 1:**
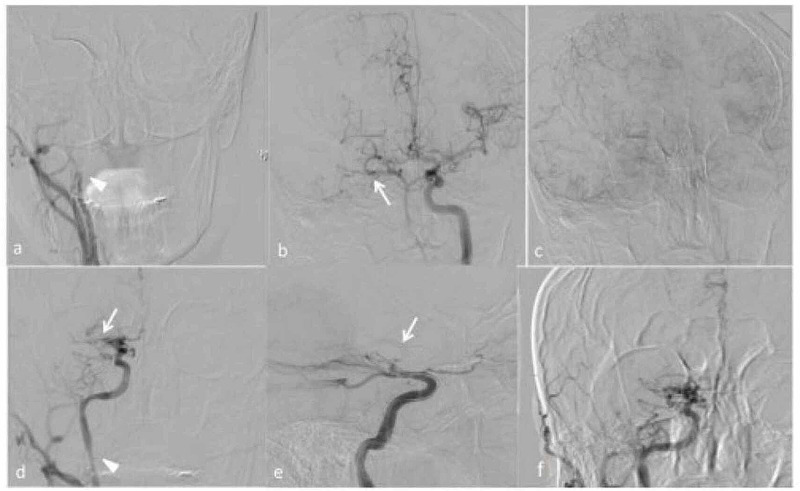
Digital subtraction angiography (DSA) a) Digital subtracted angiography (DSA) confirmed the occlusion of the right internal carotid artery (arrowhead). The DSA of the left internal carotid artery (b) showed occlusion of the M1 segment of the right middle cerebral artery (arrow) with good leptomeningeal collateral from the left side (c). Image d) showed the recanalization of the internal carotid artery with a closed-cell stent (Carotid Wallstent™; arrowhead). After four unsuccessful attempts of M1 segment recanalization with stent retriever (arrow) (e) the results were modified treatment in cerebral ischemia (mTICI) score 1 (f).

A Caucasian male patient aged between 40 and 50 years, a smoker, without other vascular risk factors, was admitted to the emergency department (ED) because of acute onset of left hemiplegia, hypoesthesia, and dysarthria. He had no known family history of cardiac, cerebrovascular, or rheumatologic disease. A non-contrast brain computed tomography (CT) was non-conclusive for either ischemic or hemorrhagic lesions. The patient was promptly centralized to our ED according to the hub-and-spoke model [1] and evaluated by a stroke specialist who revealed a neurological index of stroke scale (NIHSS) of 11. CT angiogram of head and neck was obtained, showing the right internal carotid artery (ICA) occlusion. The patient was eligible for intravenous thrombolysis with recombinant tissue plasminogen activator (r-tPA). No neurological improvement was observed after thrombolytic treatment; thus, the patient was moved to the interventional neuroradiology for endovascular approach. Computed tomography angiography (CTA) highlighted multiple artery stenosis: right internal carotid artery (ICA) and ipsilateral middle cerebral artery (MCA), anterior cerebral artery (ACA) complete occlusion, and severe left subclavian arterial stenosis. The neuroradiological picture raised concern for systemic vasculitis. A stent was placed on the right ICA, and ACA/MCA thromboaspiration was performed, with partial recovery of blood flow (Figure 1).

**Figure 2:**
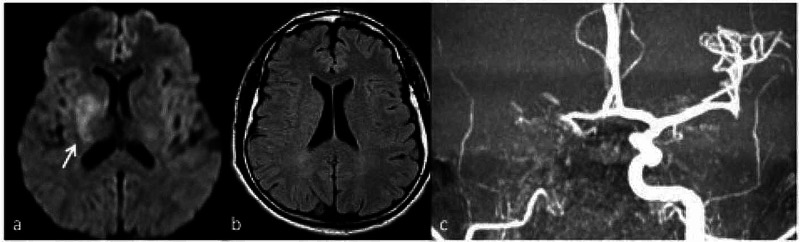
Brain magnetic resonance imaging a) Diffusion-weighted imaging, axial plan, showed a signal restriction in the right region of basal ganglia (arrow) without corresponding hyperintensity in T2w fluid attenuation inversional recovery (FLAIR) (b). The findings are consistent with acute stroke, confirmed by maximum intensity projection in coronal plan (c) of time of flight sequence showed a right tandem occlusion of internal carotid (arrowhead) and tract M1 of the middle cerebral artery (arrow).

On physical examination, the neurological deficit persisted on the left side (no effort against gravity in the left arm and leg), he had minor left facial palsy and dysarthria (NIHSS=13). A difference in blood pressure between the left and right arm (> 15 mmHg) and vascular bruit on the subclavian left artery was also detected. A brain magnetic resonance image (MRI) showed ischemic lesions involving right lenticular and basal ganglia and a right tandem occlusion of the internal carotid and tract M1 of the middle cerebral artery (Figure 2).

**Figure 3:**
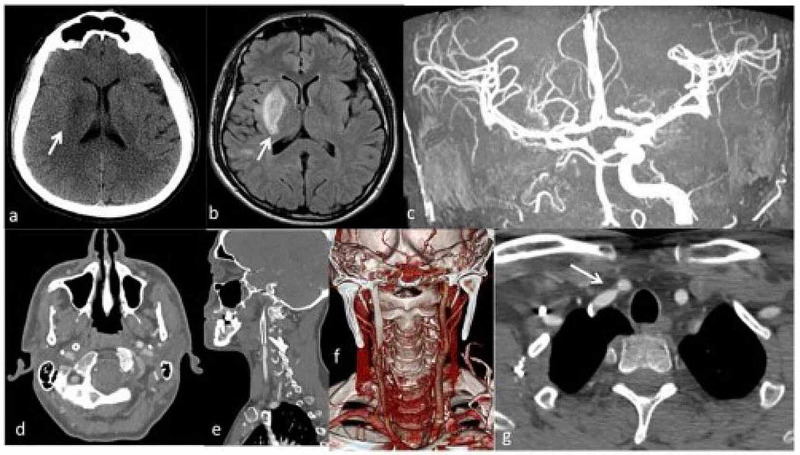
Computed tomography imaging performed after one-week follow-up The one-week follow-up computed tomography (a) showed a hypodense signal in the region of right basal ganglia (arrow) confirmed by T2w fluid attenuation inversional recovery (arrow) consistent with ischemic subacute damage. The maximum intensity projection in coronal plan (c) of time of flight sequence showed good recanalization of the right middle cerebral artery with the signal in the internal carotid artery (arrowhead). The arterial phase of computed tomography (d) confirmed the occlusion of the stent (arrowhead) showing also in the sagittal plane (e) and volume rendering imaging (f). As collateral findings, severe stenosis of the right subclavian artery (arrow) was found (g).

Laboratory analysis revealed an erythrocyte sedimentation rate (ESR) of 60 mm/h (range 0-15 mm/h), a C-reactive protein (CRP) of 2.7 mg/dl (< 1 mg/L), fibrinogen levels of 523 mg/dl (range 150-350 mg/dl). According to the American College of Rheumatology criteria, a diagnosis of TA was made [1], and the patient was started on dual antiplatelet therapy (DAPT; clopidogrel plus aspirin) and steroid therapy. A follow-up brain and neck CT was performed to control large vessels after the intravascular procedure, and an early stenting occlusion on ICA was found. As collateral findings, severe stenosis of the right subclavian artery was found (Figure 3).

Despite the stent occlusion, the patient had an excellent neurological recovery (NIHSS=0 at discharge) after an intensive neurological rehabilitation program. After a five-year follow-up, the patient was still in good clinical condition, and there was no relapse of the disease. 

## Discussion

TA is a rare disease affecting mainly young female patients presenting as an inflammatory process of large vessels such as the aorta and its main branches, with unknown etiology. TA is also described in the pediatric population. There is a wide range of manifestations from impalpable pulse or bruits, mainly interesting carotid or radial pulse, to hypertension with critical renal stenosis, myocardial ischemia with coronary involvement, or cerebrovascular diseases [2-3].

Inflammatory markers (CRP, ESR) are frequently elevated but do not correlate with disease activity. The acute phase of the disease consists of an inflammatory condition with symptoms fluctuation over weeks or months. The chronic phase of the disease is characterized by secondary symptoms resulting from vessel stenosis or occlusion [4]. The diagnostic criteria are not completely defined; indeed, angiography or magnetic resonance imaging is considered the gold standard based on vessel involvement. Type I involves the aortic arch and its branches; type II involves the thoracoabdominal aorta and its branches; type III applies when vessels of both types I and II are involved; type IV involves pulmonary arteries. Approximately 70% of patients with TA have carotid stenosis. Herein, we reported a case of TA with acute onset of ischemic stroke and multiple arterial stenosis treated with a multidisciplinary approach (intravenous thrombolysis, carotid stent placement, and clot retrieval). A retrospective study demonstrated that carotid stent placement is a feasible option for treating long segment stenosis of carotid arteries in patients with TA [5]. However, carotid stenting in TA presents an increased risk of restenosis probably because this type of intervention should be performed during the quiescent phase of the disease when the inflammatory process is still inactive [6].

Despite the stent’s occlusion, the patient had a good clinical outcome with full neurological recovery. This outcome is probably due to many factors. First of all, the timeliness of the treatment reduces neuronal death in ischemic brain tissue. Moreover, treatment with the fibrinolytic agent could be responsible for the dissolution of the small clots that incidentally reach the brain microcirculation during intravascular procedures. Finally, the chronic inflammation that features TA could allow the development of intracranial collateral circles with increased cerebral tissue resistance even in conditions of reduced blood flow. Although stroke is a rare complication in TA patients with an estimated prevalence of 11.7%, according to a recent meta-analysis [7], it could represent a catastrophic evolution of the disease overall because of the patients' young age. In our patient, the combined approach of thrombolysis and thrombectomy proved to be advantageous in clinical outcome despite the early post-procedural complication of stent closure.

## Conclusions

As the guidelines in the treatment of acute stroke associated with TA have many “grey” areas of application, and endovascular approaches are questionable due to their limited chance of success, it is advisable to evaluate the possibility of a combined treatment based on the clinical condition of the patient and the risk-benefit ratio. In this regard, the clinical experience and the multidisciplinary approach applied in our case had a pivotal role. Such an approach would eventually advocate for standardized treatment in patients with stroke and TA. 
